# Tracing the Evolutionary Pathways of Serogroup O78 Avian Pathogenic *Escherichia coli*

**DOI:** 10.3390/antibiotics12121714

**Published:** 2023-12-09

**Authors:** Eun-Jin Ha, Seung-Min Hong, Seung-Ji Kim, Sun-Min Ahn, Ho-Won Kim, Kang-Seuk Choi, Hyuk-Joon Kwon

**Affiliations:** 1 Laboratory of Avian Diseases, Department of Farm Animal Medicine, College of Veterinary Medicine and BK21 PLUS for Veterinary Science, Seoul National University, Seoul 088026, Republic of Korea; flower678@snu.ac.kr (E.-J.H.); topkin@snu.ac.kr (S.-M.H.); seungji910@snu.ac.kr (S.-J.K.); 2 Research Institute for Veterinary Science, College of Veterinary Medicine, Seoul 08826, Republic of Korea; vicky.ahn@snu.ac.kr (S.-M.A.); iamkhw52@snu.ac.kr (H.-W.K.); 3 Laboratory of Poultry Medicine, Department of Farm Animal Medicine, College of Veterinary Medicine and BK21 PLUS for Veterinary Science, Seoul National University, Seoul 088026, Republic of Korea; 4 Farm Animal Clinical Training and Research Center (FACTRC), GBST, Seoul National University, Pyeongchang 25354, Republic of Korea; 5 GeNiner Inc., Seoul 08826, Republic of Korea

**Keywords:** *Escherichia* evolution, avian pathogenic *E. coli*, *rpoB* sequence typing, network analysis, comparative genomics, CRISPR spacer, molecular prophage typing, virulence and antibiotic resistance genes, bacteriocin

## Abstract

Avian pathogenic *E. coli* (APEC) causes severe economic losses in the poultry industry, and O78 serogroup APEC strains are prevalent in chickens. In this study, we aimed to understand the evolutionary pathways and relationships between O78 APEC and other *E. coli* strains. To trace these evolutionary pathways, we classified 3101 *E. coli* strains into 306 subgenotypes according to the numbers and types of single nucleotide polymorphisms (RST0 to RST63-1) relative to the consensus sequence (RST0) of the RNA polymerase beta subunit gene and performed network analysis. The *E. coli* strains showed four apparently different evolutionary pathways (I-1, I-2, I-3, and II). The thirty-two Korean O78 APEC strains tested in this study were classified into RST4-4 (45.2%), RST3-1 (32.3%), RST21-1 (12.9%), RST4-5 (3.2%), RST5-1 (3.2%), and RST12-6 (3.2%), and all RSTs except RST21-1 (I-2) may have evolved through the same evolutionary pathway (I-1). A comparative genomic study revealed the highest relatedness between O78 strains of the same RST in terms of genome sequence coverage/identity and the spacer sequences of CRISPRs. The early-appearing RST3-1 and RST4-4 prevalence among O78 APEC strains may reflect the early settlement of O78 *E. coli* in chickens, after which these bacteria accumulated virulence and antibiotic resistance genes to become APEC strains. The zoonotic risk of the conventional O78 APEC strains is low at present, but the appearance of genetically distinct and multiple virulence gene-bearing RST21-1 O78 APEC strains may alert us to a need to evaluate their virulence in chickens as well as their zoonotic risk.

## 1. Introduction

Avian pathogenic *E. coli* (APEC) is an extraintestinal pathogenic *E. coli* (ExPEC) that causes severe economic loss in the poultry industry and increases public health concerns [1,2]. The different compositions and linkages of carbohydrates determine the O serogroups of *E. coli*, and to date, 185 O serogroups have been identified [3]. The high diversity of O serogroups in *E. coli* strains may be partially due to escape mutations and selection resulting from the detrimental infection of lytic bacteriophages, with O serogroup changes occurring through various mechanisms, including the homologous recombination of newly introduced O-antigen gene clusters (O-AGCs) [3]. Although the frequencies of O serogroups of APEC strains depend on farms and countries, O78 is one of the most frequent serogroups in the world [4,5].

Various molecular methods, such as multilocus sequence typing (MLST), pulsed-field gel electrophoresis (PFGE), phylogrouping, *rpoB* sequence typing (RSTing), molecular prophage typing, and profiling spacer sequences of the CRISPR–Cas (clustered regularly interspaced short palindromic repeats and CRISPR-associated genes) system, have been applied to differentiate *E. coli* strains and elucidate the molecular epidemiology of outbreaks of pathogenic *E. coli* infections [6,7,8,9]. Core genome-based MLST (cgMLST) is applied to elucidate evolutionary relationships between *E. coli* strains, other *Escherichia* species, and *Salmonella enterica* serovars, but the strategy of using multiple genes with different evolutionary statuses may complicate the analysis of the results [10]. Pangenome analyses have provided new insights into the evolution of species, antibiotic resistance, and the pathogenicity of bacteria [11]. However, simpler and more cost-effective first-line methods are needed.

The RNA polymerase beta subunit (RpoB) is an enzymatic component of RNA polymerase. RNA polymerase plays biologically and evolutionarily important roles, including pivotal roles in decoding gene information into mRNAs for protein translation and the transcription of other important RNAs. Therefore, RpoB is one of the key molecules in which the evolutionary histories of the present united DNA/RNA/protein worlds are embedded. The applicability of the *rpoB* gene as a chronological molecule to understand the progenitor–progeny relationships of bacteria was hinted at in *Staphylococcus aureus* (*S. aureus*) strains [12,13]. Humans are natural hosts of *S. aureus*, and the bacterium spills over into other animals via adaptation based on missense mutations in essential genes and the acquisition/loss of genes [14,15,16,17]. The major human strains have only two (*rpoB* sequence type, RST 2-1) or four (RST4-1) mutations compared to the consensus *rpoB* sequence, which is a hypothetical progenitor sequence. Although the *rpoB* genes of the RST2-1 and RST4-1 strains have not changed considerably from the consensus sequence (RST0), they have acquired various prophages and genomic islands to increase genome sizes. Therefore, the commensal life of *S. aureus* in humans may not cause the accumulation of multiple mutations in *rpoB*. In this context, the presence and predominance of early-appearing RSTs in certain hosts may reflect the early settlement of bacterial species in terms of their evolution. Currently, *Shigella* species are considered toxigenic variants of *E. coli*, and *E. fergusonii* and *E. albertii* are pathogenic and are not easily differentiated from *E. coli* due to biological similarities [18,19,20]. Additionally, *Salmonella enterica* is genetically closely related to *E. coli* [21]. While *E. coli* genomes are subject to changes in size and gene contents, some host-adapted *Salmonella* genomes are suppressed to such modifications by other genetic circuits and by the robustness and redundancy of the genomic network, making them less variable [22,23,24]. Accordingly, a trial to understand the progenitor–progeny relationship between *E. coli* and other *Escherichia* and *Salmonella* species may be valuable. Therefore, a *rpoB*-based approach to unravel the genetic relationships among them may be of interest.

Due to the similarity between APEC and human ExPEC, such as uropathogenic *E. coli* and neonatal meningitis *E. coli*, the zoonotic risks of APEC need to be addressed. Genomic relatedness between O1 APEC and human ExPEC has been suspected, but the zoonotic risk of O78 APEC still needs to be further determined [25,26]. The cumulative acquisition of virulence and antibiotic-resistance genes in pathogenic *E. coli* increases public health concerns and needs to be elucidated more clearly.

In this study, we modified our previous RSTing approach for *E. coli* similarly to the *S. aureus* RSTing scheme [6,12]. We performed network analysis with single nucleotide polymorphisms (SNPs) of representative RSTs, including *Shigella* strains and selected strains of other *Escherichia* species and *Salmonella enterica*. We typed the RSTs of O78 APEC strains recorded in Korea during 2012–2020 and characterized their virulence, antibiotic resistance, and microcin/colicin gene contents. Furthermore, we performed a comparative genomics analysis of the identified major RSTs of O78 APEC strains to demonstrate the evolutionary relationships between them and other *E. coli* strains of the same and different RSTs.

## 2. Results and Discussion

### 2.1. O78 APEC Strains Identified during 2012–2020

A total of 31 of the O78 APEC strains accounted for 19.0% of the 163 APEC strains identified during 2012–2020, and they were isolated from birds of various ages and poultry types: broiler breeders (45.2%), commercial layers (32.3%), broilers (19.4%), and Korean native chickens (3.2%). Phylogenetic group B1 was most predominant (51.6%), followed by C (25.8%), E (9.7%), F (9.7%), and A (3.2%) (Table 1).

### 2.2. Modified rpoB Sequence Typing (RSTing) of E. coli Strains

In this study, we modified our previous RSTing scheme by reclassifying RSTs according to the number of nucleotide differences from the consensus sequence (RST0) [6,12]. A total of 3101 *E. coli* strains, comprising 3029 sourced from the GenBank database and an additional 32 O78 APEC strains (including the previously reported E123 strain) along with 40 APEC strains of other serotypes (in manuscript preparation), were classified into 306 RSTs, spanning from RST2-1 to RST63-1 [6]. The predominant RSTs identified were RST3-1, accounting for 17.4%, followed by RST6-1 at 12.7%, among others (Table S1). The thirty-one O78 strains were classified into RST4-4 (45.2%), RST3-1 (32.3%), RST21-1 (12.9%), RST4-5 (3.2%), RST5-1 (3.2%), and RST12-6 (3.2%), and E123 was classified as RST3-1 (Table 1). We collected O78 reference strains using BLAST searches with the nucleotide sequence of the O-AGC of the E123 strain, and their RSTs and serotypes are summarized in Table S2. The twenty O78 reference strains were classified into RST3-1 (70%), RST6-1 (15%), RST4-30 (5%), RST7-3 (5%), and RST21-1 (5%), and including our 32 O78 strains, RST3-1 (48.1%, 25/52) was most prevalent, followed by RST4-4 (26.9%, 14/52) and RST21-1 (9.6%, 5/52).

*Shigella* species are now considered *E. coli* sensu lato and are thought to have evolved in a convergent manner by acquiring Shiga toxin genes [21]. Some *Shigella* strains are classified into *E. coli* RSTs, including RST3-1, but others are classified into the unique *Shigella* RSTs listed in Table S3. The results may reflect the early and continuous acquisition of *Shigella* phenotypes during *E. coli* evolution. Among 306 RSTs, 68 RSTs (22.2%) possessed missense mutations, and O78 strains of RST4-30 (Y47C) and RST12-6 (T141S-F195L-V196A-L204P-L213V-K422R) possessed different mutations from rifampin resistance-related mutations (500-575 and V146F/W) (Table S1) [27]. The majority of *E. coli* strains among the early RSTs, RST2-1, and RST3-1, originated from humans, e.g., 67.1% of RST2-1 strains (Tables S4 and S5).

### 2.3. Hypothetical Evolutionary Pathways of E. coli and O78 APEC Strains

**Figure 1:**
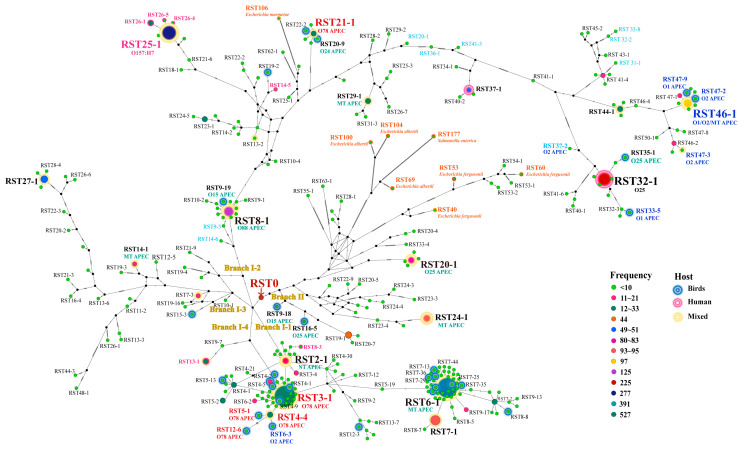
Median-Joining Network of *E. coli* RSTs. The evolutionary tracing of a total of 3029 *E. coli* strains was conducted based on *rpoB* SNP haplotypes. This study also includes representatives of other species within the genus *Escherichia* as well as the genus *Salmonella*, which are labeled using orange text. The median-joining network method was employed for this analysis, and the network was constructed using popART software (v.1.7) [29,30]. RST numbers were derived from the consensus sequence (RST0), ranging from RST2-1 to RST63-1. Two main branches were identified: branch I, comprising subbranches 1-4, and branch II. RST3-1 and RST4-4 were located within subbranch I-1, while RST21-1 was classified under subbranch I-2. The frequency of RSTs was reflected by variations in the colors and sizes of the circles within the network. A blue border indicates that the hosts of the RSTs are birds, a red border signifies human hosts, and a yellow border represents a mixed host category that includes the environment, humans, and other animals. Black dots are median vectors that can be biologically interpreted as extant unsampled sequences or extinct ancestral sequences [29].

Using the SNPs of RSTs, network analysis was performed to understand the evolutionary pathways of *E. coli*, *E. fergusonii*, *E. albertii*, *E. marmotae*, and *S. enterica* serovar Indiana (*S*. Indiana) (Figure 1). Two branches, I and II, came from RST0, and branch I was divided into four subbranches: I-1, I-2, I-3, and I-4. Subbranch I-1 contained prevalent RST3-1, RST6-1, and all of the O78 RSTs except for RST21-1. The farthest RST from RST0 in subbranch I-1 was RST13-7. Branch I-2 contained the O78 strain containing RST21-1, and the farthest RST was RST62-1. Interestingly, an *E. marmotae* strain (RST106) emerged from branch I-2. Branch I-3 contained RST27-1, and the farthest RST was RST48-1. Branch I-4 was short and contained only RST9-1 and RST13-1. Branch II contained prevalent RST24-1 and RST20-1, and the farthest RST was RST63-1. Interestingly, *E. albertii* (RST69, RST100, and RST104), *E. fergusonii* (RST40, RST53, and RST60), and *S*. Indiana (RST177) came from branch II. Considering the low RST numbers and the identity percentages (98.5–99.1%) of *E. fergusonii* strains, they may be more closely related to *E. coli* strains than other *Escherichia* species. *E. fergusonii* and *E. albertii* are emerging pathogens and are difficult to differentiate from *E. coli* [18]. Therefore, the present RSTing scheme may help to identify them. *Salmonella enterica* is known to be most closely related among the different bacterial genera, and our result showing the evolutionary pathway sharing of *S*. Indiana and some *E. coli* strains may be interesting to study further [21,28]. We also performed phylogenetic analysis using nucleotide sequences of *rpoB* utilizing Bayesian inference (Figure S1). The clustering pattern and topology are similar to network analysis, but network analysis intuitively shows progenitor and progeny relationships between RST0 and other RSTs.

The *rpoB* gene sizes of major gram-positive and gram-negative bacteria were compared, and the RSTs of additional bacteria with the same size of *rpoB* as *E. coli* are summarized in Table 2. The *rpoB* gene sizes of the gram-positive bacteria are smaller than those of the gram-negative bacteria and increase in order from 3531 bp to 3717 bp. In the case of gram-negative bacteria, the lengths of *rpoB* varied from shorter (4026 bp) to longer (4239 bp) than those of *E. coli*. Thus, the differences in the nucleotide sequences and the length of *rpoB* may provide a hint of the evolutionary direction and order of important pathogenic bacteria.

### 2.4. General Genomic Information of the Six Representative O78 APEC Strains from RST3-1, RST4-4, and RST21-1

The *E. coli* pangenome is highly variable, and the reference genome sequences of O79 APEC strains are not sufficient for resequencing. For these reasons, the complete genome sequences of the six representative O78 APEC strains classified into the three major RSTs, aRST3-1 (E19057 and E123), RST4-4 (E18005 and E19025), and RST21-1 (E12049 and E14033), were determined using de novo sequencing and compared with the corresponding reference strains, which were selected on the basis of an identical serogroup (PSUO78), high genome coverage/identity (NCTC11129), or both (APEC O78). Basic information on the genomes, such as their lengths, GC contents, numbers of CDSs, rRNAs, tRNAs and CRISPRs, coding ratios (%), plasmids, and numbers of genomic islands, is summarized in Table 3. The determined genome sizes ranged from 4,885,187 bp (E19057) to 5,170,367 bp (E12049), and the GC ratio was 50.60–50.79%. The H antigen type, sequence type (ST) of MLST, and molecular prophage type (mPPT) of O78 strains were determined, and within the six strains, two CRISPRs, between 1 and 4 plasmids, and 69 and 94 genomic islands were identified (Table 3).

### 2.5. Correlation of Genomic Sequence Coverage/Identity and CRISPR Contents between the Same RSTs Containing Major O78 Strains

**Figure 2:**
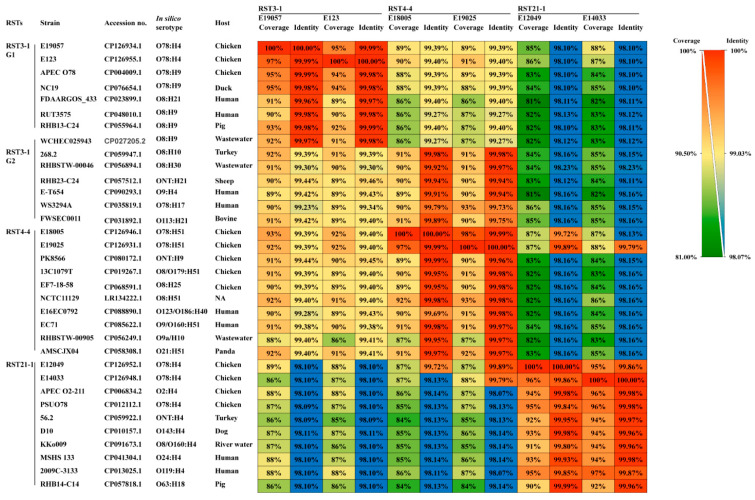
Comparison of genomic coverages (%) and identities (%) of O78 *E. coli* strains. A heatmap was constructed to represent the average nucleotide identity (ANI) and alignment coverage between O78 APEC and other RST strains. The values are visually depicted based on color intensity, with the alignment coverage ranging from red to green and the nucleotide identity represented by a color gradient from red to blue.

The whole-genome sequences of O78 strains were compared, and the coverage and nucleotide identities were measured. The coverage/identity between the same RSTs of the O78 APEC strains was highest, and the coverage/identity values of RST3-1, RST4-4, and RST21-1 were 97%/99.99%, 98%/99.99%, and 96%/99.98%, respectively (Figure 2). It is reasonable to deduce that the higher the genomic coverage/identity is, the closer the evolutionary distance.

Not all *E. coli* strains within the same RST exhibited a high degree of genomic similarity; some strains exhibited higher genetic congruence, while others showed less or much less genetic similarity. The early-appearing RSTs, such as RST2-1 and RST3-1, encompassed more than three (RST2-1_G1, G2, G3, etc.) and four (RST3-1_G1, G2, G3, G4, etc.) distinct genome coverage/identity groups, respectively (Figure 2; Tables S4 and S5). However, some strains showed relatively lower genome coverages, ranging from 87 to 93%, with high genome sequence identities ranging from 99.91 to 99.97% (RST2-1 G1) (Table S4). As the number of compared *E. coli* genomes increased, the number of core genes decreased to 753 [11]. The pangenome of *E. coli* is regarded as open and still evolving by gene acquisition and diversification [31]. The lower genome coverage may be due to the different accessory and unique genes that were acquired from different metagenomic environments surrounding the habitats of different *E. coli*. Therefore, the comparison of genome coverages/identities may be a simple way to evaluate evolutionary relatedness between strains before core and pangenome analyses. Interestingly, the RST21-1 O78 APEC strains revealed elevated genome identities (99.72–99.89%) exclusively with RST4-4 APEC strains but not with other RST4-4 strains from different countries (Figure 2). Therefore, recent genetic exchanges between them are suspected (Figure S2).

**Table 3:** Comparative genomics of the representative O78 APEC strains.

	E19057	E123	APEC O78	E18005	E19025	NCTC11129	E12049	E14033	PSUO78
Accession no.	CP126934.1	CP126955.1	CP004009.1	CP126946.1	CP126931.1	LR134222.1	CP126952.1	CP126948.1	CP012112.1
Origin	Chicken	Chicken	Turkey	Chicken	Chicken	unknown	Chicken	Chicken	Chicken
RST	3-1	3-1	3-1	4-4	4-4	4-4	21-1	21-1	21-1
MLST (Achman)	ST23	ST369	ST23	ST155	ST155	ST155	ST11749	ST48	ST5940
mPPT	2-5	2-3-4	3-4	1-2-3-5(2)-6	1-2-3(2)-4-5-6	2-3-4-5	1-2-5	1-2-4-5	2-4-5-7
Serotype	O78:H4	O78:H4	O78:H9	O78:H51	O78:H51	O8, O60:H51	O78:H4	O78:H4	O78:H4
Total Length (bp)	4,885,187	4,998,625	4,798,435	5,129,598	5,086,337	5,008,027	5,170,367	5,021,334	4,988,493
GC Content (%)	50.60%	50.64%	50.70%	50.64%	50.70%	50.78%	50.74%	50.79%	50.80%
No. of CDSs	4586	4690	4504	4771	4751	4697	4788	4658	4791
No. of rRNA	22	22	19	21	22	22	22	22	22
No. of tRNA	86	82	88	92	95	89	97	90	88
No. of CRISPRS	2	2	2	2	2	2	2	2	2
Coding ratio (%)	87.30%	87.10%	87.60%	87.00%	87.20%	87.10%	86.80%	87.20%	86.60%
Plasmid 1 ^a^	pEND_Eco19057-1 (CP126935.1) (IncI1-1 (alpha), 100%; 110,406 bp)	pEND_Eco 123-1 (CP126956.1) (IncFIB, 98.39%; 121,788 bp)	217,830 bp	pEND_Eco 18005 (CP126947.1) (IncFIB, 98.39%; 204,838 bp)	pEND_Eco 19025-1 (CP126932.1) (IncFIB, 98.39%; 160,439 bp)	No data	pEND_Eco 12049-1 (CP126953.1) (IncFIB, 99.12%; 155,857 bp)	pEND_Eco 14033-1 (CP126949.1) (IncFIB, 98.39%; 145,212 bp)	pPSUO78_1 (CP012113.1) (132,464 bp)
Plasmid 2	pEND_Eco 19057-2 (CP126936.1) (IncFIB, 98.39%; 104,962 bp)	pEND_Eco 123-2 (CP126957.1) (No hit; 68,426 bp)	113,260 bp		pEND_Eco 19025-2 (CP126933.1) (IncY, 99.38%; 90,722 bp)		pEND_Eco 12049-2 (CP126954.1) (IncI1-1(alpha), 100%; 114,229 bp)	pEND_Eco 14033-2 (CP126950.1) (IncI1-1 (alpha), 100%; 115,173 bp)	pPSUO78_2 (CP012114.1) (109,613 bp)
Plasmid 3								pEND_Eco 14033-3 (CP126951.1) (IncFIB (H89-PhagePlasmid), 98.7%; 108,542 bp)	
Plasmid 4								pEND_Eco 14033-4 (CP130557.1) (IncI2(delta), 97.15%; 61,041 bp)	
No. Genomic islands (GI) ^b^	69	70	47	94	88	61	94	77	80

^a^ Plasmid classification according to [32,33]. ^b^ Prediction was performed using the Islandviewer 4 (including IslandPick, IslandPath-DIMOB, SIGI-HMM, and Islander).

**Figure 3:**
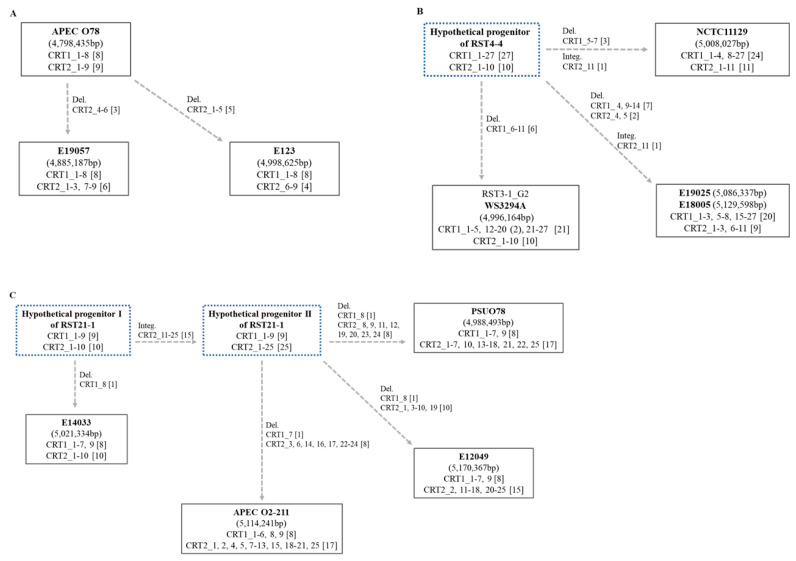
Evolutionary model based on the spacer sequences of CRISPRs in O78 APEC strains according to RST: (**A**) RST3-1, (**B**) RST4-4, and (**C**) RST21-1. The CRISPR spacers of genetically related strains of each RST were compared, and their progenitor–progeny relationships were hypothesized in terms of spacer contents. In the case of RST3-1 strains, E19057 and E123 may be different progenies of APEC O78 and may have lost different numbers of spacers in CRT2. In the case of RST4-4, E19025, E18005, and NCTC11129 may have lost different numbers of spacers in CRT1 and CRT2 of a hypothetical progenitor. A RST3-1_G2 strain, WS3294A, shares the same hypothetical progenitor of RST4-4, reflecting their evolutionary relatedness. Only RST4-4 strains E19025, E18005, and NCTC11129 acquired one spacer (CRT2_11). In the case of RST21-1, E14033 may be a progeny of hypothetical progenitor I, and APEC O2-211, PSU-O78, and E12049 are progenies of hypothetical progenitor II. All the RST21-1 strains may have experienced different deletions in CRT1 and CRT2.

The contents of CRISPR spacers reflect the recent invasion–defense history of *E. coli* strains, and we can obtain information about evolutionary relatedness between *E. coli* strains and the identities of present invaders by comparing the contents and numbers of spacers [34]. The compositions of CRISPR spacers tend to be conserved among *E. coli* strains with identical RSTs, especially when their genome sequence coverages/identities are notably high (Tables S5 and S6). Typically, a new spacer is appended to the 5′-end to the most recent spacers, and some unnecessary spacers are lost randomly [8]. To elucidate the evolutionary trajectory of spacers, considering both the nucleotide sequences and length, we collected all the spacers of the closely related *E. coli* strains of each RST and inferred the evolutionary progression based on the presence and absence of specific spacers. As a result, the stepwise deletion and/or integration of spacers among related *E. coli* strains within RST3-1, RST4-4, and RST21-1 could be delineated by considering the spacer profiles of putative progenitors (Figure 3). For RST3-1, E19057 and E123 may have lost spacers 4-6 and 1-5 in CRT2, respectively. In the case of RST4-4, there may be a hypothetical progenitor harboring all spacers, and the RST4-4 strains may have lost varying spacers. However, in E19025, E18005, and NCTC11129, a new spacer was added. The evolutionary steps of RST21-1 spacers were more complicated, and two hypothetical progenitors were included. Hypothetical progenitor I evolved into hypothetical progenitor II by adding 15 spacers. E14033 lost one spacer of hypothetical progenitor I, but E12049, PSUO78, and APEC O2-211 lost multiple spacers of hypothetical progenitor II. Therefore, E14033 was closer to the recent progenitor of RST21-1 strains than others in terms of spacer evolution. At present, the available data are not sufficient to answer it. It is reasonable to ask why most strains tend to delete spacers. The spacers of representative RST3-1, RST4-4, and RST21-1 strains targeted plasmids and bacteriophages. In contrast to the RST3-1 spacers, mainly targeting plasmids (93.3%), the RST4-4 (100%) and RST21-1 (90.0%) spacers mainly targeted bacteriophage genes (Table S7). In a previous study, the targets of spacers were chromosomal regions (12%), plasmids (31%), and phages (57%), and the bias of the targets of RST3-1 strains toward plasmids and RST4-4 and RST21-1 toward phages observed in the present study was unexpected [34].

### 2.6. Evolutionary Linkage between Early-Appearing RSTs

**Figure 4:**
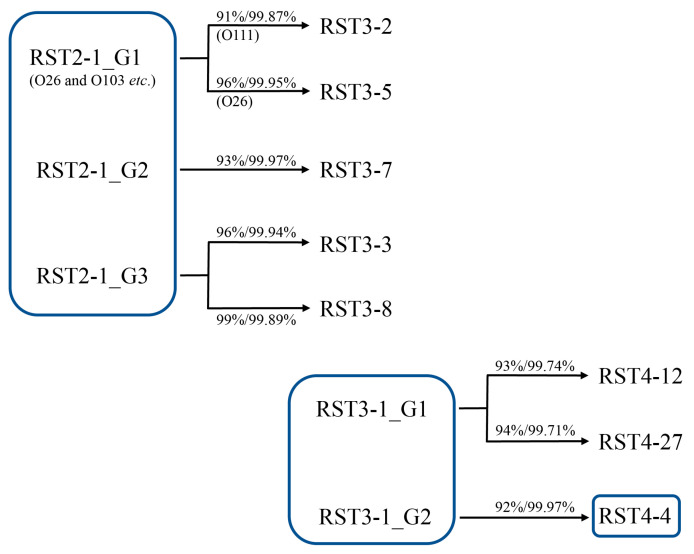
Evolutionary relationships of RSTs appearing early. *E. coli* lineages ranging from RST2-1 to RST 4-27 were predicted using the measure of genomic coverage (%)/identity (%).

Genome types RST2-1_G1 (O26), RST2-1_G2, and RST2-1_G3 exhibited high genomic coverage/identity related to RST3-2 (91/99.87%, O111)/3-5 (96/99/95%, O26), RST3-7 (93/99.97%), and RST3-3 (96/99.94%)/3-8 (99/99.89%), respectively (Figure 4). The RST3-1 O78 APEC strains were classified under RST3-1_G1 and showed high genomic coverages/identities and CRISPR spacer contents related to non-O78 and O8 human strains of the same genome type (Table S5). They showed higher genomic coverage/identities in relation to RST4-12 (93/99.74%) and RST4-27 (94/99.71%) (Figure 4). No genetic linkage was observed between the RST2-1 and RST3-1 strains, and they may have evolved independently of each other. RST3-1_G2 displayed relatively high genomic coverage/identity (92/99.97%) and shared some CRISPR spacers with RST4-4 O78 APEC strains (Figure 4; Table S5).

### 2.7. Evolution of O Serogroups in Different RSTs

On the basis of genome sequence coverage/identity and RSTs, we hypothesized the evolution of O serogroups. The O antigens of simpler structures and components may appear earlier than more complex O antigens and much simpler O serogroups may appear in earlier RSTs [35]. The major O serogroup of RST2-1 is O26, which is composed of linearly linked trisaccharides without side branches [→3)-L-Rha-(α1→4)-L-FucNAc-(α1→3)-DGlcNAc-(β1→] [3]. The genome sequence coverages/identities between O26 strains are 96–99% and 99.96–99.99%. However, other strains with different O antigen structures, such as linearly linked tetrasaccharides (O71, O118, and O186) and pentasaccharides (O69 and O103), show high genome sequence coverages/identities (97–98%/99.97–99.98%) related to O26 strains (Table S4). The O antigen structures and O-AGC sequences apparently differ between them, and the different O-AGCs may have been horizontally transferred to the genomes of the prevalent O26 strains [3,36,37]. In contrast to clearly different O157:H7 EHEC strains (RST25-1), the evolutionary links between O26, O103, and O111 ETEC strains were obscure, but the high genomic coverage and identity observed between RST2-1 O26 ETEC strains and other RST2-1 O103 and RST3-2 O111 strains can explain the parallel evolution of various O serogroup ETEC strains (Figure 4) [38].

The prevalence of the O8 serogroup among RST3-1_G1 strains may reflect the early appearance of the O8 antigen with a simple structure and components, [→3)-D-Man-(β1→2)-D-Man-(α1→2)-D-Man-(α1→], during *E. coli* evolution. Most RST3-1_G1 strains originated from healthy or clinical human specimens and may have evolved in humans. O78 serogroup RST3-1_G1 strains were only present in chickens and turkeys, and they showed more complex mPPTs than human non-O78 RST3-1_G1 strains (0 to 2 prophages) (Table S5). This may support the possibility that the O78 serogroup appeared in chickens and evolved via the acquisition of more prophages in the intestines of poultry. The O serogroups of RST3-1_G2 were mixed with various serogroups, including O8 and O78. The only RST3-1_G2 human-origin O78 strain, WS3294A, was ETEC, which contained a more complex and unique mPPT (4-6-7-11) than other RST3-1 O78 strains (Table S2). The close genetic linkage observed between non-O78 strains of RST3-1_G2 and RST4-4 O78 strains may suggest the acquisition of O78-AGC by some strains within RST3-1_G2 from RST3-1_G1 or RST4-4 O78 strains. No O78 RST4-4 strains, except for Korean APEC strains, have been identified to date (Table S9). The RST3-1 O78 APEC strain AH01 was classified as RST3-1_G3 and is the only O78 avian strain in the genome type. In the RST3-1_G4 genome type, three O78 strains (ATCC 43896, L3_E36, and 00-3279) were identified, and some of them were pathogenic and showed high genomic coverage/identity (98–99%/99.99%) and similar mPPTs to each other (Table S2). However, their mPPTs were different from those of other RST3-1 avian O78 strains in terms of the presence of prophage 7. The presence of a relatively simple O78 antigen, consisting of four linearly linked carbohydrates of two different components (D-mannose and D-GlcNAc) in RST3-1 and RST4-4 may support the early appearance of the O78 antigen during *E. coli* evolution (Figure 2). The distribution of the O78 serogroup in other RSTs in branch I-1 can be explained by the maintenance of the O78 serogroup during the evolution of *rpoB*.

The O78 serogroup appeared abruptly among RST21-1 strains in branch I-2. The RST21-1 strains showing high genomic coverage/identity and common CRISPR spacer contents had variable O serogroups, and most of the O serogroups contained more evolved structures, except O78 (Table S8). The majority of RST21-1 strains (45.8%, 11/24) originated from avian hosts and belonged to the non-O78 serogroup, except for the PSUO78 strain. Some still possessed simpler mPPT types (2-5, 2-7, and 2-4-5). Therefore, RST21-1 O78 APEC strains may have evolved from non-O78 RST21-1 strains through the acquisition of the prevalent O78-AGC in avian hosts.

Thus, we found several new cases of an O serogroup shift, such as the case of O157:H7 *E. coli* originating from O55 *E. coli* in RST25-1, identified based on the RSTing scheme and simple genomic coverages/identities [39]. Although the RST3-1, RST4-30, and RST6-1 O78 ETEC strains have been reported in humans, the relatively low zoonotic risk of major O78 APEC strains can be supported by the low frequencies of O78 *E. coli* strains in clinical human cases at present (Table S2).

### 2.8. Profiling Virulence Genes of O78 APEC Strains

The presence of 24 representative virulence genes grouped into six categories was confirmed (Table 1 and Table S10). All O78 strains were found to possess the *fimH*, *crlA*, *iss, sitA, iroN, hlyF*, *ompT*, and *malX* genes. Additionally, the following genes were detected at various frequencies: *fyuA* (90.6%), *iutA* (87.5%), *irp2* (87.5%), *mat* (75.0%), *hra* (75.0%), *tsh* (75.0%), *traT* (68.8%), and *ireA* (65.6%). As previously reported, the redundant possession of siderophores and the absence of toxin genes such as *stx1*, *stx2*, *lt*, and *st* were verified [5]. The virulence gene content varied among strains, ranging from 9 to 20, and there was an evident accumulation of multiple virulence genes (Table 1). Additionally, the accumulation orders and patterns of virulence genes are hinted at in our results (Table S10). In the case of adhesion genes, *fimH* and *crlA* may have been acquired first, after which *hra* and *mat* were acquired by the majority of O78 strains (75.0%, 24/32). Subsequently, *papC* or *iha* were optionally acquired. Siderophore genes may have acquired *sitA/iroN* followed by *fyuA*, *iutA/irp2*, *ireA*, and *chuA*. Regarding toxin genes, *hlyF* may have been acquired first, followed sequentially by *tsh*, *pic/vat,* and *astA*. The distribution of virulence genes on the chromosome and/or plasmids was determined using the genome sequences of the E19057, E123, E18005, E19025, E12049, and E14033 strains, and the results are summarized in Figure S3. All of the adhesion genes, some siderophore genes (*fyuA*, *irp2*, *ireA*, and *chuA*), some toxin genes (*pic*, *vat,* and *astA*), one invasion gene (*tia*), and *malX* were present only on the chromosome. *iss*, *sitA,* and *ompT* were redundantly present on the chromosome and plasmid. *traT*, *iroN*, *iutA*, *hlyF*, and *tsh* were present only on the plasmids, and the origin sequences of multiple virulence gene-bearing plasmids were classified under the same type, IncFIB.

### 2.9. Antibiograms of O78 APEC Strains

The resistance of the strains to 24 antibiotics of eight classes and their resistance genes are summarized in Table S11. Among multidrug-resistant (MDR) strains, the frequency of RST types reveals that RST4-4 is the most predominant at 61.1%. This is followed by RST3-1 at 16.7% and RST21-1 at 11.1%. Within each RST group, the prevalence of MDR strains was as follows: RST3-1 at 27.3%, RST4-4 at 78.6%, and RST21-1 at 50%. The prevalence of multidrug resistance (MDR) in the RST4-4 strains was observed to be significantly elevated in comparison to the RST3-1 strains (*p* < 0.05), as determined through the application of the Mann–Whitney U test (Table 4). In particular, MDR RST4-4 and MDR RST21-1 strains also possess many virulence genes that need to receive special attention.

The antibiotics tested in this study associated with the highest resistance were amoxicillin (100%), enrofloxacin (65.6%), oxytetracycline (56.3%), cefazolin (50.0%), streptomycin (28.1%), sulfamethoxazole/trimethoprim (21.9%), and florfenicol (6.3%). There was no strain showing resistance against colistin (Table S11). The recorded resistance rates are similar to other reports in Korea [4,40]. During 1980-2005, resistance to streptomycin and tetracycline was highest among Korean APEC strains, reaching 84.2%, followed by resistance to enrofloxacin (71.3%) and ampicillin (67.3%) [41]. The maintenance of antibiotic resistance is costly when the associated resistance mechanism is specific, and the decreased resistance to tetracycline and streptomycin in this study may be somehow related to the decreased usage of these antibiotics in chicks on poultry farms. However, high resistance frequencies of ampicillin and enrofloxacin still revealed no effect of the efforts to reduce antibiotic abuse in the field. Resistance to florfenicol is low at present, but it has become the preferred antibiotic for the treatment of APEC in the field. Therefore, changes in the resistance and prevalence of the *floR* gene need to be monitored. The resistance to cephalosporins and the acquisition of related resistance genes observed during 2014–2019 may be related to the recent increase in ceftiofur usage for the *in ovo* inoculation of embryonated chicken eggs for bacterial clearance in Korea, as in other countries [42,43]. However, it is noteworthy that the reduction in the use of certain antibiotics, such as tetracyclines and penicillins, did not necessarily lead to a decrease in antibiotic-resistant bacterial populations. In some cases, there was even a marginal increase observed. These findings suggest a complex interplay of factors influencing the persistence and evolution of antibiotic resistance, extending beyond the simple dynamics of antibiotic presence or absence [24,40,41,42,43].

**Figure 5:**
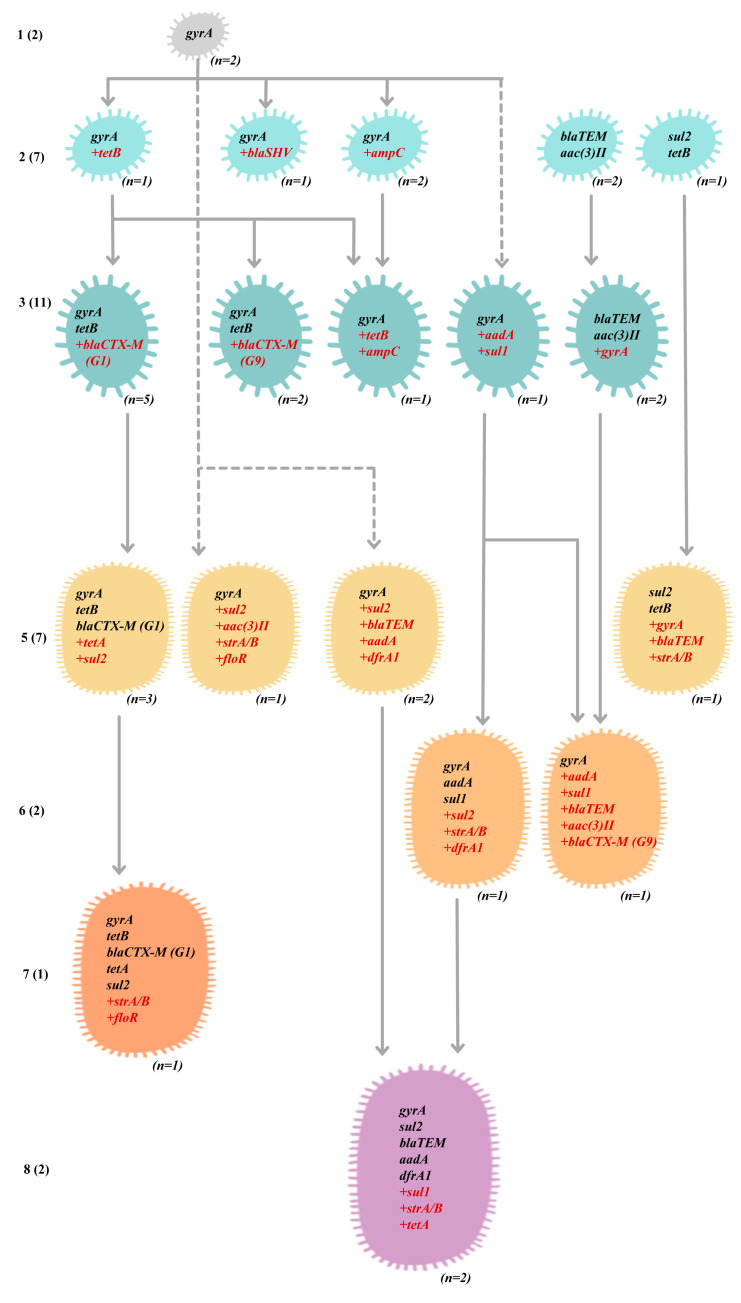
Theoretical interconnections between O78 APEC strains (n = 32) with different contents of antibiotic resistance genes (genotypes). This schematic illustrates relationships between different genotypes.

Resistance to penicillins was associated with *bla_TEM_* (31.3%), *bla_SHV_* (3.1%), and PBP3 mutations (D350N and S357N), and extended resistance to cephalosporins was associated with *bla_CTX__-1_* (28.1%), *bla_CTX-9_* (9.4%), and *ampC* (6.3%). Quinolone resistance is related to single or multiple mutations in *gyrA*, *gyrB*, and/or *parC*, and the S83L mutation of *gyrA* provides intermediate resistance to enrofloxacin and is shared by 90.6% of strains. For complete resistance to quinolones, additional mutations, such as the D87N of *gyrA* or the S492N of *gyrB,* were needed. Resistance to aminoglycosides was associated with *strA/strB* (21.9%), *aadA1/A2* (18.8%), and *aac(3)II* (18.8%). Resistance to tetracyclines was associated with *tetB* (50.0%) and *tetA* (18.8%). Resistance to sulfonamides was associated with *sul2* (37.5%) and *sul1* (15.6%), and resistance to trimethoprim was associated with *dfrA1* (12.9%). Resistance to sulfamethoxazole/trimethoprim discs (SXT) was determined by *dfrA1* in combination with *sul1* or *sul2*. Resistance to phenicols was associated with *floR* (6.3%). Only class 1 and 2 integrons were present in seven and one strains among the tested O78 APEC strains (Table 5 and Table S11). The locations of resistance genes are summarized in Figure S3. The presence of class 1 integrons in E12049 and E14033 may be related to the presence of *aadA1/sul1* genes in similar plasmids in terms of size and origin sequence type, IncI1-1α (Table 3 and Table S11) [41,44]. Thus, the genotypic and phenotypic antibiograms matched each other well in the present study. Based on a comparison of the contents and numbers of resistance genes of O78 APEC strains, the different genotypes are depicted and theoretically interconnected in Figure 5. Most O78 APEC strains (21/32) shared a minimal *gyrA* gene mutation (S83L) and possessed 19 different combinations of *tetA*, *tetB*, *ampC*, *bla_TEM_*, *bla_SHV_*, *bla*_CTX-M_ groups 1 and 9, *sul1*, *sul2*, *dfrA1*, *aadA*, *aac(3)II*, *strA/B*, and *floR*. Therefore, some genotypes can be interconnected via the acquisition or deletion of resistance genes. Although we addressed specific genes related to antibiotic resistance, other unknown genes and cryptic mechanisms may be involved. In the case of MDR strains, it is very common that the resistance mechanisms are nonspecific and, therefore, are associated with mechanisms related to transport, cellular homeostasis or cellular aggregation, and a decrease in the area/volume ratio. Additionally, we need to consider the combinatorial capacity of the gene regulatory network to optimize resistance phenotypes [24,45,46,47,48,49].

### 2.10. Profiles of Microcins and Colicins of O78 APEC Strains

To date, various microcins and colicins have been reported among *E. coli* strains [50,51,52]. First, we conducted an in silico analysis to screen for their presence in six Korean O78 APEC strains (Table 6). Microcin V, microcin J25, microcin H47, colicin Ia, colicin Ib, and colicin M were present on the chromosomes or plasmids. Except for E123 and E14033, all others possessed double or triple bacteriocins. We screened the presence of the above bacteriocins using PCR for the remaining O78 strains, and the results are summarized in Table S12. The frequency of microcin V (78.1%) was the highest, followed by colicin M (65.6%), microcin J25 (53.1%), colicin Ib (53.1%), microcin H47 (15.6%), and colicin Ia (3.1%).

We tested the bacteriocin activity of each O78 APEC strain against DH5a and six representative O78 APEC strains using a modified spot assay. Except for E16023, all the other O78 strains showed inhibitory activity with different intensities (from + to ++++). RST3-1 strains, E123 and E19057, were highly susceptible to bacteriocin produced by 11 (34.4%) and 5 (15.6%) of the O78 strains in comparison with RST21-1 E14033 (6.3%) and RST4-4 E19025 (3.1%). Among the twelve O78 strains killing at least one O78 strain, RST4-4 strains were more frequent (18.8%, 6/32; 42.9%, 6/14 RST4-4) than the others, including RST3-1 (9.4%, 3/32; 27.3%, 3/11 RST3-1), RST21-1 (6.3%, 2/32; 50.0%, 2/4 RST21-1), and RST12-6 (3.1%, 1/32) strains. A significant difference was observed in the number of the six bacteriocins assessed between RST3-1 and RST4-4 (*p* < 0.05), as determined using the Kruskal–Wallis test. (Table S12). In contrast to other strains killing one or two O78 strains, the RST4-4 strains E15016 and E15025 killed three strains (RST3-1 E123, E19057, and RST21-1 E14033) (Table S13). The development of relatively broad colicidal activity among RST4-4 strains may be a cause of the prevalence of RST4-4 compared with other RSTs.

## 3. Materials and Methods

### 3.1. Bacterial Strains and Identification

Thirty-one O78 APEC strains from 2012 to 2020 and a previously reported O78 APEC strain (E123) were isolated from poultry cases consigned to our laboratory (Table 1). Lactose-nonfermenting colonies on the MacConkey agar (BD Difco, Becton Dr, NJ, USA) were identified using MALDI (matrix-assisted laser desorption ionization)–TOF (time-of-flight) mass spectrometry (Bruker, Billerica, MA, USA) and the bacterial species were further confirmed using 16S rRNA sequencing [53]. Bacteria were cultured in Luria–Bertani (LB) broth (Duchefa, Netherlands) at 37 °C and preserved in 20% glycerol LB broth stocks at −60 °C until use.

### 3.2. Serotyping and Phylogrouping of E. coli

The O serogroup of APEC was determined using an agglutination test, employing commercial O78-specific antisera in accordance with the manufacturer’s protocol (Denka Seiken, Tokyo, Japan). Additionally, PCR was performed utilizing serogroup-specific primers, as described in a previous study [54]. In silico serotyping of O and H antigens was carried out utilizing SerotypeFinder (v.2.0.1, https://cge.food.dtu.dk/services/SerotypeFinder/) accessed on 1 May 2023 in conjunction with EcoSP (http://ecosp.dmicrobe.cn/) software [55,56] To identify the phylogenetic group, a multiplex PCR assay developed by Clermont was applied following the described protocol [57].

### 3.3. rpoB Sequence Typing (RSTing) and Molecular Prophage Typing (mPPTing)

We refined a previously reported *rpoB* sequence type (RST) by implementing some modifications. The PCR assay and sequencing were conducted as in a previous study [6].

The complete *rpoB* nucleotide sequences of *E. coli* (3029 strains) and other bacteria were collected from GenBank (National Center for Biotechnology Information, NCBI) (up to 5 April 2023) (Table 2). Upon aligning the whole *rpoB* sequences, including those of O78 APEC with MEGA X (v.11.0.13) [58], haplotypes were determined based on SNPs using DnaSP (v.6.12.03) [59]. Initial RST numbers were assigned by counting SNPs in the *rpoB* sequences, and subsequent sub-numbers were serialized according to the distinct haplotypes. The *rpoB* network was constructed using the median-joining network method with the aid of popART (v.1.7) [29,30]. Bayesian phylogenetic trees were constructed using MrBayes (v.2.2.4) [60], implemented in Geneious Prime (v.2023.2.1) (https://www.geneious.com, Auckland, New Zealand). Labels for the figures were edited using Adobe Illustrator 2023 (Adobe Inc., San Jose, CA, USA).

In silico molecular prophage typing was performed as previously reported, with a modification to add information about the copy numbers of each mPPT [7].

### 3.4. Molecular Pathotyping

The presence of 34 virulence genes in O78 APEC strains was investigated using 34 primers based on a combination of a 5-set multiplex PCR and four single PCR assays, as reported previously (Table S14) [61,62,63,64,65,66].

### 3.5. Antibiotic Susceptibility Testing and Molecular Profiling of Antibiotic Resistance Genes

Sensitivity to various antibiotics listed in supplementary Table S11 was assessed utilizing the disk diffusion method, according to Kirby and Bauer [67], in conjunction with the guidelines provided by the Clinical and Laboratory Standards Institute (CLSI) [68]. For the determination of colistin susceptibility, the broth microdilution method (BMD) was employed as described in previous studies [69,70] and in accordance with the EUCAST guidelines (www.eucast.org, Basel, Switzerland).

Subsequently, 37 antimicrobial resistance genes were amplified from all O78 APEC isolates using PCR and utilizing the primers listed in Table S14, as previously reported [71,72,73,74,75,76,77,78,79,80,81,82,83,84,85,86,87,88]. Mutations in the quinolone resistance-determining regions (QRDRs) of *gyrA*, *gyrB*, *parC*, and *parE* were identified via sequencing after PCR, as reported previously [76,77].

### 3.6. Comparative Genomics

Whole-genome sequencing (WGS) was performed using de novo assembly and PacBio sequencing technology (PacBio) after constructing a SMRTbell long-read library. To ensure accurate genome sequences, error correction was applied utilizing a TruSeq DNA library (insert size: 350 bp) in conjunction with Illumina high-quality data (100 bp paired-end). DFAST was utilized for gene annotation [89]. MLST 2.0. and PubMLST were applied to match *E. coli* MLST alleles [90,91]. CRISPRs and *cas* genes in the genome were detected using CRISPRCasFinder (v.1.1.2), and the matching of spacer sequences was accomplished with the CRISPRTarget tool along with GenBank data using BLASTN [92,93,94]. The visualization of genomic analysis was performed using the BLAST Ring Image Generator (BRIG) (Figure S2) [95].

### 3.7. Identification of Bacteriocin Genes in O78 APEC

The primers utilized for molecular detection of bacteriocins in this study are described in Table S14 [50]. The *E. coli* cells were cultured overnight in LB broth, and the bacterial DNA was obtained by lysing the cells at 95 °C for 10 min. Aliquots of 20 μL of a reaction mixture containing 2 μL DNA, 2 μL primer set, and 16 μL DW were added to the Maxime PCR PreMix strips (i-StarTaq) kit (iNtRon, Seongnam, Republic of Korea). The amplification conditions were as follows: 1 cycle at 95 °C for 5 min, 30 cycles of 95 °C for 30 s, 55 °C for 30 s, and 72 °C for 1 min, and a final cycle of 5 min at 72 °C, followed by a hold at 12 °C.

### 3.8. Antimicrobial Activity Test of Bacteriocin

Brief screening for sensitivity to bacteriocin from O78 APEC was conducted using a modified spot assay method. Bacterial broth cultures were diluted in PBS to achieve a turbidity of 0.1–0.2 MacFarland, ensuring similar numbers of seven indicator *E. coli* strains (DH5α, E19057, E123, E18005, E19025, E12049, and E14033). Three milliliters of the diluent was evenly spread onto square LB agar plates (SPL, Pocheon, Republic of Korea) and allowed to dry until no liquid remained. Overnight bacterial cultures of 32 O78 APEC strains and DH5α in LB broth were concentrated 100-fold, and a 10 μL volume of the fluid was spotted onto the indicator lawn plates. After overnight incubation at 37 °C, the sensitivity to O78 bacteriocin was determined based on the thickness of clear zones around the spotted sites: -, no inhibition zone; +, 1–2 mm; ++, 3–4 mm; +++, 5–6 mm; ++++, ≥7 mm.

### 3.9. Statistical Analysis

Fisher’s exact tests were used to determine the significant association between the prevalence of RST and MDR strains. The Mann–Whitney U test was utilized to compare the MDR frequencies among the RST groups. Additionally, the presence of distinct virulence genes and bacteriocins across different RST groups was analyzed using the Kruskal–Wallis test, followed by the Dunn–Bonferroni correction. All statistical analyses were conducted using SPSS (v.26.0) (IBM Corp., Armonk, NY, USA), and the results were interpreted with 95% confidence intervals. To ensure reliability, all experiments in this study were repeated at least twice.

## 4. Conclusions

Taken together, the RSTing and network analysis in the present study provided new insights into the evolutionary relationships between *E. coli* and other related bacteria. The major RSTs, RST3-1 and RST4-4, of O78 serogroup *E. coli* may have settled down and adapted to poultry to become APEC by accumulating virulence and antibiotic resistance genes. The zoonotic potential of O78 APEC strains is not currently high, but the prevalence of MDR RST4-4 and the appearance of new RST21-1 O78 APEC strains bearing multiple virulence genes encourage the continuous monitoring of their prevalence and virulence in poultry and their zoonotic risk.

## Figures and Tables

**Table 1:** O78 avian pathogenic *E. coli* strains characterized in this study.

Strain	Year of Isolation	Chicken Type ^a^	Age (Day-Old)	Phylo- Group	RST ^b^	No. of Virulence Genes	Strain	Year of Isolation	Chicken Type ^a^	Age (Day-Old)	Phylo- Group	RST	No. of Virulence Genes
E12049	2012	KNC	40	F	21-1	20	E16015	2016	B	24	B1	5-1	15
E14033	2014	L	76	F	21-1	19	E17039	2017	BB	263	B1	12-6	15
E12090	2012	L	124	F	21-1	18	E19033	2019	BB	134	B1	4-4	15
E18005	2018	BB	209	B1	4-4	17	E19057	2019	L	148	C	3-1	14
E16018	2016	L	451	B1	4-4	16	E16023	2016	BB	203	E	3-1	14
E18016	2018	BB	163	B1	4-4	16	E16039	2016	L	252	B1	4-4	14
E18027	2018	B	2	E	21-1	16	E19024	2019	L	315	C	3-1	14
E19014	2019	BB	7	B1	4-4	16	E20019	2020	L	310	E	3-1	14
E19025	2019	B	7	B1	4-4	16	E17001	2017	BB	264	C	3-1	13
E19034	2019	BB	235	B1	4-4	16	E12050	2012	B	26	C	3-1	12
E19045	2019	L	19	B1	4-4	16	E13028	2013	BB	29	C	3-1	12
E19070	2019	BB	170	B1	4-4	16	E20042	2020	L	161	C	3-1	11
E12091	2012	BB	236	B1	4-4	15	E19013	2019	BB	77	C	4-5	9
E15016	2015	B	30	B1	4-4	15	E19040	2019	BB	195	C	3-1	9
E15026	2015	BB	273	B1	4-4	15	E19056	2019	B	3	A	3-1	9
E16011	2016	L	136	B1	4-4	15	E123	2003	NA ^c^	NA	C	3-1	17

^a^ L: layer chicken; B: broiler chicken; BB: broiler breeder chicken; KNC: Korean native chicken. ^b^ RST: *rpoB* sequence type. ^c^ NA, not available.

**Table 2:** Comparison of *rpoB* gene size, identity, and *rpoB* sequence typing.

Pathogenic Bacteria	Strain	Accession No.	Size of *rpoB*	RST	Identity (%)
*Corynebacterium diphtheriae*	31A	CP003206.1	3531	ND	ND
*Bacillus anthracis*	A2084	NC_007530.2	3534	ND	ND
*Staphylococcus aureus*	PMB81	CP03444.1	3552	ND	ND
*Listeria monocytogenes*	EGD-e	NC_003210.1	3555	ND	ND
*Streptococcus pneumoniae*	Hu17	NZ_CP020549.1	3612	ND	ND
*Enterococcus faecalis*	T5	NZ_KB944666.1	3615	ND	ND
*Clostridium perfringens*	CPI 18-6	NZ_CP075979.1	3705	ND	ND
*Clostridium difficile*	s-0253	NZ_CP076401.1	3717	ND	ND
*Vibrio cholerae*	RFB16	CP043554.1	4026	ND	ND
*Escherichia coli*	RST0	ND ^a^	4029	0	100
*Escherichia fergusonii*	RHB10-C04	CP057918.1	4029	40	99.1
*Escherichia fergusonii*	ATCC 35471	CP042945.1	4029	53	98.7
*Escherichia fergusonii*	RHB02-C14	CP055872.1	4029	60	98.5
*Escherichia albertii*	RM10507	CP043258.1	4029	69	98.3
*Escherichia albertii*	ChinaSP140150	CP025676.1	4029	100	97.5
*Escherichia albertii*	BIA_36	CP117590.1	4029	104	97.4
*Escherichia marmotae*	W49-2	CP093239.1	4029	106	97.4
*Salmonella enterica* serovar Indiana	SI67	CP050783.1	4029	177	95.6
*Salmonella bongori*	N268-08	CP006608.1	4029	251	93.8
*Klebsiella pneumoniae*	XH210	CP052761.1	4029	259	93.6
*Enterobacter cloacae*	colR/S	CP010512.1	4029	264	93.4
*Serratia marcescens*	SmUNAM836	CP012685.1	4029	491	87.8
*Yersinia pseudotuberculosis*	IP2666pIB1	CP032566.1	4029	588	85.4
*Yersinia pestis*	FDAARGOS_603	CP033690.1	4029	590	85.4
*Proteus mirabilis*	1035	CP072779.1	4029	758	81.1
*Pasteurella multocida*	FDAARGOS_218	CP020405.2	4029	1027	74.5
*Haemophilus influenzae*	Hi375	CP009610.1	4032	ND	ND
*Pseudomonas aeruginosa*	PAO1	AE004091.2	4074	ND	ND
*Acinetobacter baumannii*	Ab421_GEIH-2010	CP014266.1	4074	ND	ND
*Legionella pneumophila*	C9	CP015941.1	4107	ND	ND
*Bordetella bronchiseptica*	NCTC10543	LR134326.1	4113	ND	ND
*Neisseria gonorrheae*	AT159	CP097846.1	4179	ND	ND
*Moraxella catarrhalis*	CCRI-195ME	CP018059.1	4239	ND	ND

^a^ ND, not determined.

**Table 4:** Frequency of RSTs among MDR O78 APEC strains (*n* = 18).

	RST3-1	RST4-4	RST5-1	RST12-6	RST21-1
Frequency out of MDR strains (%) ^a^	3/18	11/18	1/18	1/18	2/18
	(16.7%)	(61.1%) ^b^	(5.6%)	(5.6%)	(11.1%)
MDR Frequency out of each RST (%)	3/11	11/14	1/1	1/1	2/4
	(27.3%)	(78.6%)	(100%)	(100%)	(50%)

^a^ Significant correlation between RST and MDR (*p* < 0.05). ^b^ Significant difference between RST3-1 and RST4-4 (*p* < 0.05).

**Table 5:** Frequency of antibiotic resistance-related genetic markers of O78 APEC strains (*n* = 32 including the E123 strain).

Antibiotics Resistance- Related Genes	*gyrA*	*tetB*	*sul2*	*bla_TEM_*	*bla_CTX-M_* *_-G1_*	*strA/strB*	*tetA*	*aadA1* */A2*	*aac(3)II*	*sul1*	*dfrA1*	*bla_CTX-M_* *_-G9_*	*ampC*	*floR*	*bla_SHV_*
Frequency (%)	29	16	12	10	9	7	6	6	6	5	4	3	2	2	1
	(90.6)	(50.0)	(37.5)	(31.3)	(28.1)	(21.9)	(18.8)	(18.8)	(18.8)	(15.6)	(12.5)	(9.4)	(6.3)	(6.3)	(3.1)

**Table 6:** Contents of microcins and colicins in the representative RSTs.

Strain	RST	Chromosome	Plasmid				No. of Microcin/Colicin ^a^
			1	2	3	4	
E123	3-1	None	Microcin V (Colicin V)	None			1
E19057	3-1	None	Colicin Ia	Microcin V (Colicin V)			2
E18005		microcin H47	Microcin J25				4
			Colicin M				
	4-4		Microcin V (Colicin V)				
			Colicin B (pseudo)				
E19025		None	Microcin J25	None			3
			Colicin M				
	4-4		Microcin V (Colicin V)				
			Colicin B (pseudo)				
E12049	21-1	None	Colicin M	Colicin Ib			2
E14033	21-1	None	None	ColIcin Ib	None	None	1

^a^ Counted numbers exclude colicin B (classified as pseudogenes).

## Data Availability

The WGS data for the O78 APEC chromosome and plasmids are available in the NCBI database under BioProject PRJNA978016. Accession numbers of the individual genomes are described in Table 3.

## Supplementary Materials

The following supporting information can be downloaded at: https://www.mdpi.com/article/10.3390/antibiotics12121714/s1, Figure S1: Bayesian phylogenetic analysis of *E. coli rpoB* sequence types; Figure S2: Comparison of genome sequences of O78 APEC strains; Figure S3: The location of virulence genes and antibiotic resistance genes in the O78 APEC genome; Table S1: *rpoB* sequence types (RSTs) of *E. coli* strains; Table S2: O78 *E. coli* strains among the analyzed *E. coli* strains; Table S3: RSTs of *Shigella* species; Table S4: In silico genetic characterization of RST2-1 *E. coli* strains; Table S5: In silico genetic characterization of RST3-1 *E. coli* strains; Table S6: Comparison of CRISPR spacers of the representative O78 *E. coli* strains; Table S7: Targets of CRISPR spacers of the representative O78 APEC strains; Table S8: In silico genetic characterization of RST21-1 *E. coli* strains; Table S9: In silico genetic characterization of RST4-4 *E. coli* strains; Table S10: Profiles and frequency of virulence genes of O78 APEC strains; Table S11: Genotypic and phenotypic correlations of antibiotic resistance among O78 APEC strains; Table S12: Profiles of bacteriocins and colicins among O78 APEC strains; Table S13: Colicin activity of O78 APEC strains; Table S14: Primer sets used in this study.
