# Supplementary material for: Tracing the Evolutionary Pathways of Serogroup O78 Avian Pathogenic Escherichia coli
Source: Antibiotics (Basel). 2023 Dec 9;12(12):1714. doi: 10.3390/antibiotics12121714 (PMC10740950; doi:10.3390/antibiotics12121714)
Supplement: Supplementary file 1 [file antibiotics-12-01714-s001.zip › Supplementary figures S1-S3_revision.pdf]

Supplementary Figures

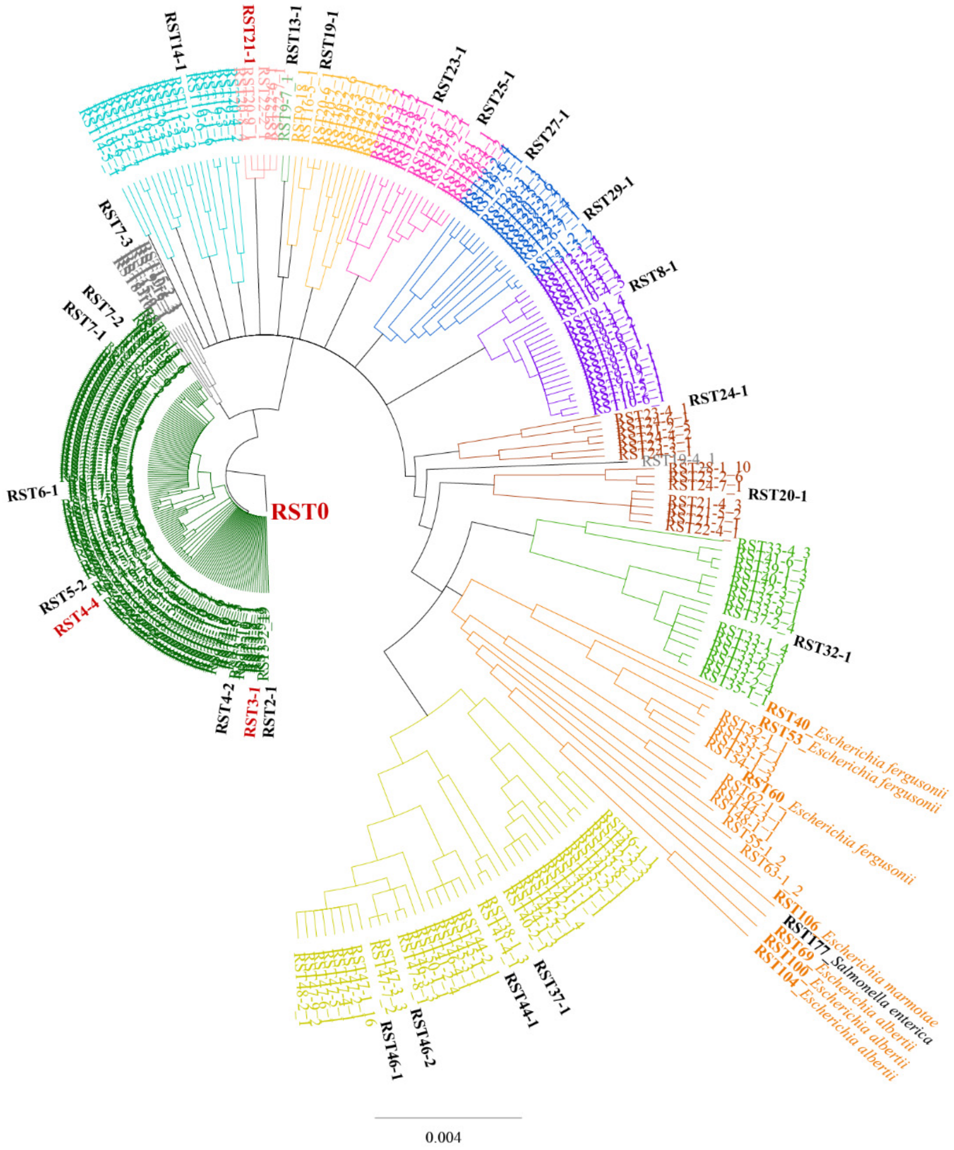

Figure S1. Bayesian phylogenetic analysis of *E. coli rpoB* sequence types presented as a circular tree, constructed using MrBayes V.2.2.4. The tree was rooted using RST0 as the outgroup (consensus sequence). A dark green section corresponds to Branch I-1 from the network analysis shown in Figure 1. Predominantly prevalent RST numbers are highlighted in bold.

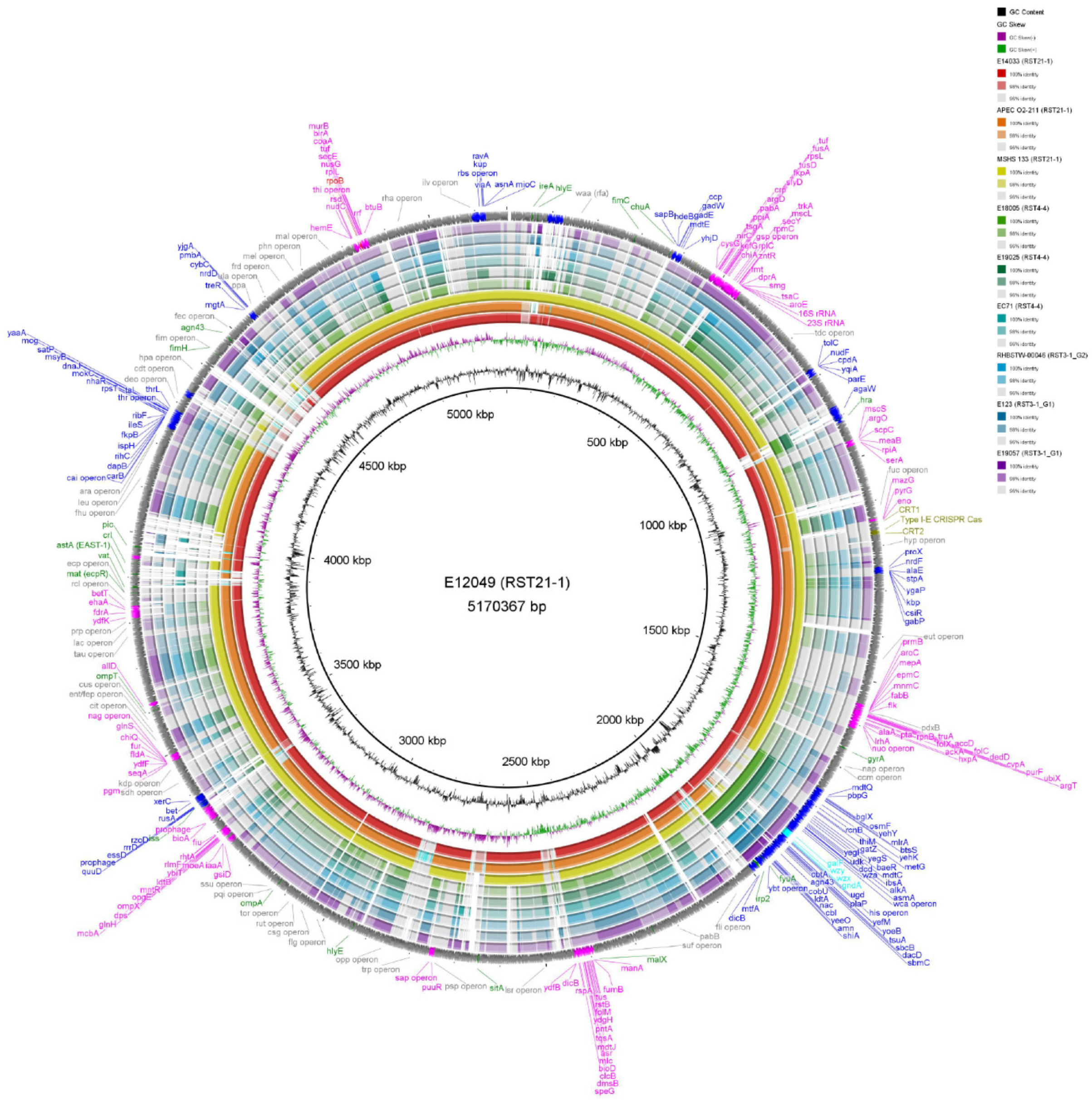

Figure S2. Comparison of genome sequences of O78 APEC strains. Regions of high genomic identity between RST21-1 (E12049 and E14033) isolated from chickens and RST4-4 were highlighted in blue. High identity between RST3-1_G2 and RST4-4 were marked in pink. Virulence genes and antibiotic resistance genes present in the E12049 genome are denoted in green, while CRISPR-Cas systems in the E12049 genome are indicated in yellow.

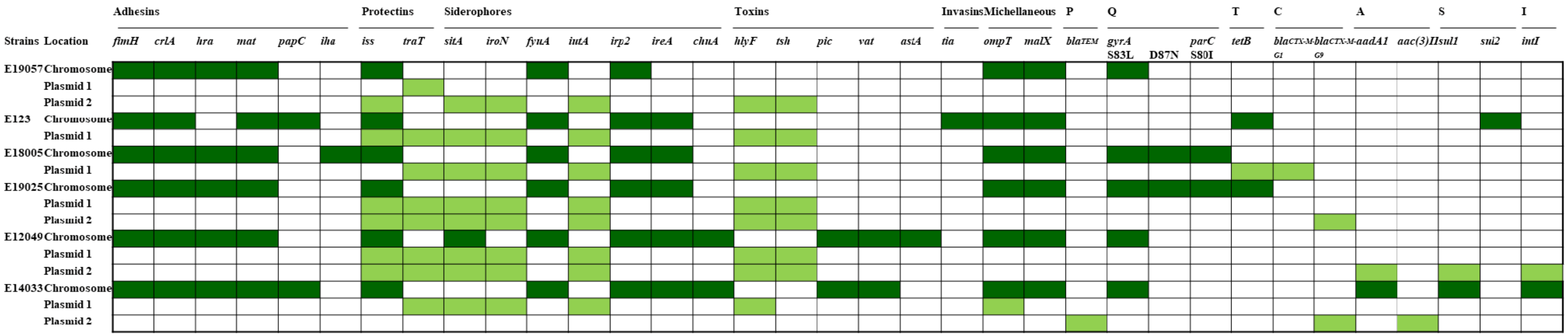

Figure S3. The location of virulence genes and antibiotic resistance genes in the O78 APEC genome is depicted in the diagram. Dark green signifies genes located in chromosomal DNA, while light green represents those carried on plasmid DNA. P, penicillins; Q, quinolones; T, tetracyclines; C, cephalosporins; A, aminoglycosides; S, sulfonamides; I, integron.
